# Caveolin-1 rs4730751 single-nucleotide polymorphism may not influence kidney transplant allograft survival

**DOI:** 10.1038/s41598-019-52079-8

**Published:** 2019-10-29

**Authors:** Mehdi Maanaoui, Rémi Lenain, Aghilès Hamroun, Cynthia Van der Hauwaert, Benjamin Lopez, Jean-Baptiste Gibier, Marie Frimat, Grégoire Savary, Benjamin Hennart, Romain Larrue, Nicolas Pottier, Franck Broly, François Provôt, Marc Hazzan, François Glowacki, Christelle Cauffiez

**Affiliations:** 1 Service de Néphrologie, CHU Lille, F-59000 Lille, France; 2 University Lille, EA4483, F-59000 Lille, France; 3 Service d’Immunologie, CHU Lille, F-59000 Lille, France; 4 Institut de Pathologie, CHU Lille, F-59000 Lille, France; 5 University Lille, INSERM UMR1172, F-59000 Lille, France; 6 University Lille, INSERM UMR995, F-59000 Lille, France; 7 Service de Toxicologie et Génopathies, CHU Lille, 59000 Lille, France; 8 Département de la Recherche en Santé, CHU Lille, F-59000 Lille, France

**Keywords:** Nephrology, Genetics research

## Abstract

Caveolin-1 is a protein (encoded by the *CAV1* gene) supposedly harboring a protective effect against fibrosis. *CAV1* rs4730751 single nucleotide polymorphism (SNP) AA genotype was initially associated with lower graft survival compared to non-AA. However, subsequent studies could not find the same effect. *CAV1* rs4730751 SNP was investigated on 918 kidney donors. Multivariate Cox-model analyses were performed to evaluate risk factors for graft loss. Longitudinal changes on long-term estimated glomerular filtration rate (eGFRs) were evaluated with a linear mixed model. Histopathological findings from protocolled biopsies after 3 months post transplantation were also analyzed. Donor *CAV1* rs4730751 genotyping proportions were 7.1% for AA, 41.6% for AC and 51.3% for CC. The AA genotype, compared to non-AA, was not associated with lower graft survival censored or not for death (multivariate analysis: HR = 1.23 [0.74–2.05] and HR = 1.27 [0.84–1.92]). Linear mixed model on long-term eGFRs revealed also no significant difference according to the genotype, yet we observed a trend. AA genotype was also not associated with a higher degree of fibrosis index on protocolled kidney biopsies at 3 months. To conclude, donor *CAV1* rs4730751 SNP may impact on kidney transplantation outcomes, but this study could not confirm this hypothesis.

## Introduction

Genomic studies are unraveling the genetic architecture of complex diseases and traits, and evidence is emerging that genetic information might be clinically relevant in some scenarios. In the field of kidney transplantation, several genetic polymorphisms such as those affecting *APOL1*^1^, *ABCB1*^2–4^, *CYP3A4*^5–7^ or *CYP3A5*^8–10^, have been shown to influence allograft outcome. Moreover, the importance of the donor’s genetic make-up was largely overlooked. Caveolin-1 has recently gained interest with the discovery of one *CAV1* Single Nucleotide Polymorphism (SNP) associated with allograft failure^11^. Caveolin-1 is the primary structural component of caveolae, involved in endocytosis and cell signaling^12^. It is ubiquitously expressed, especially in the kidney, from glomerular to epithelial cells^13^. As the lipid-raft caveolae contribute to TGFβ receptor degradation pathway, and thus decrease TGFβ signaling^14^, Caveolin-1 exerts a protective effect on fibrosis^15^, a pathological feature occurring post-transplantation^16^. Moore and colleagues were the first team which identified a significant association between *CAV1* rs4730751 SNP and a higher risk of allograft failure (donor AA versus AC and CC: HR = 1.77 [1.08–2.90])^11^. Analysis of kidney biopsies from grafts that had failed revealed a higher degree of fibrosis in the group of patients harboring an AA-genotype graft. Interestingly, the rs4730751 SNP is an intronic variant that has not been found to be in linkage disequilibrium with other exonic variants likely to alter Caveolin-1 protein function^11^. Thus, the precise roles of this SNP and its functional consequences have not been uncovered so far.

This seminal study has led to the evaluation of *CAV1* SNPs involvement in various diseases, such as chronic kidney diseases^17^, pancreas transplantation^18^, Anti-Neutrophilic Cytoplasmic Autoantibody (ANCA) vasculitis^19^ or cancers^20,21^. However, the enthusiasm has been somewhat tempered by the controversies that have risen about the real impact of *CAV1* SNPs in the field of kidney transplantation. Indeed, Ma and colleagues found opposite results, as the screening of 16 *CAV1* SNPs (including rs4730751) in 1233 kidney transplants could not reproduce Moore’s observations^22^. Recently, graft survival was also not associated with *CAV1* rs4730751 SNP either from donors or recipients in two other cohorts^23,24^.

Hence, considering these uncertainties, we carried out a study in a large-scaled cohort in order to evaluate the impact of donor *CAV1* rs4730751 SNP on kidney transplantation outcomes, using a combined analysis of graft survivals, long-term estimated Glomerular Filtration rates (eGFRs) and histopathological data from systematic kidney biopsies.

## Results

### Study population and baseline characteristics

From the 1^st^ of January 2000 to the 31^st^ of December 2016, 918 donors for kidney transplantation were genotyped for the *CAV1* rs4730751 SNP. Alleles A and C were in equilibrium according to the Hardy-Weinberg law (respectively p = 0.27 and q = 0.73). *CAV1* rs4730751 AA, AC, and CC genotypes were observed in respectively 7.1% (n = 65), 41.6% (n = 382), and 51.3% (n = 471) of donors. All donors and recipients’ demographical characteristics are summarized in Table 1. There was no difference between AA and non-AA donors, or between their respective recipients. Median follow-up was 47.7 months (23.7–119.1).

**Table 1:** Baseline donors and recipients characteristics according to *CAV1* AA and non-AA genotype.

Characteristics	non AA (n = 853)	AA (n = 65)	*p* value
Donor sex, male (versus female)	556 (65.2)	39 (60.0)	0.48
Donor age: mean (SD)	48.8 (16.2)	47.7 (16.6)	0.59
BMI donor: mean (SD)	26.0 (5.3)	25.1 (3.5)	0.052
Cause of death			0.63
Stroke	415 (48.7)	30 (46.2)	
Trauma	291 (34.1)	20 (30.8)	
Anoxia	119 (14.0)	12 (18.5)	
Other	28 (3.3)	3 (4.6)	
Cold ischemia time (minutes): mean (SD)	1136 (399)	1136 (430)	0.99
Recipient sex, male (versus female)	532 (62.4)	42 (64.6)	0.82
Recipient age: mean (SD)	50.3 (13.1)	52.7 (14.4)	0.19
BMI recipient: mean (SD)	24.9 (4.5)	24.3 (4.5)	0.29
Number of previous grafts			0.59
0	700 (82.1)	54 (83.1)	
1	127 (14.9)	11 (16.9)	
2	23 (2.7)	0 (0.0)	
3	3 (0.4)	0 (0.0)	
Cause of ESRD			0.09
Diabetes	72 (8.4)	9 (13.8)	
Glomerulonephritis	281 (32.9)	26 (40.0)	
Tubulo-interstitial	285 (33.4)	17 (26.2)	
Vascular	46 (5.4)	5 (7.7)	
Others	50 (5.9)	0 (0.0)	
Unknown	119 (14.0)	8 (12.3)	
Number of HLA mismatch (HLA A, B, DR, DQ): mean (SD)	3.8 (1.2)	3.8 (1.2)	0.86

Except where indicated otherwise, values were the number (%). SD = standard deviation, BMI = Body Mass Index, ESRD = End-stage renal disease, HLA = Human Leukocyte Antigen.

### Graft outcomes: survival estimates and Cox models

**Figure 1:**
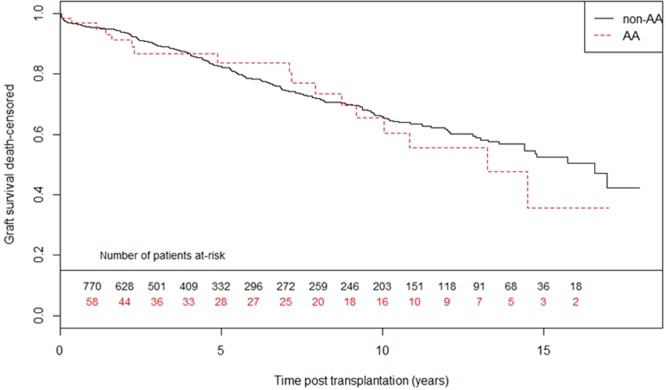
Kaplan-Meier estimates for graft-survival censored for death: *CAV1* rs4730751 single nucleotide polymorphism AA versus non-AA. Log-rank test: p = 0.63.

Using the Log-Rank test (Fig. 1), no long-term difference was observed for graft survival - censored for death (GS-DC), nor for graft survival - non-censored for death (GS-DNC), between AA and non*-*AA genotype (p = 0.64 and p = 0.64 respectively). Using a Cox model for multivariate analysis (Table 2), an AA genotype was not significantly associated in uni- or multivariate analyses with GS-DC or GS-DNC (multivariate analysis: HR = 1.23 [0.74–2.05] and HR = 1.27 [0.84–1.92] respectively).

**Table 2:** Multivariable Cox model for graft survival.

Variable	GS-DC				GS-DNC			
	Univariable	*p* value	Multivariable	p value	Univariable	*p* value	Multivariable	p value
*CAV1* genotype AA (versus non AA)	1.12 [0.68–1.85]	0.644	1.23 [0.74–2.05]	0.423	1.10 [0.73–1.66]	0.639	1.27 [0.84–1.92]	0.265
Donor age (per 10 years)	1.24 [1.13–1.36]	<0.001	1.41 [1.25–1.60]	<0.001	1.31 [1.21–1.42]	<0.001	1.30 [1.18–1.44]	<0.001
Donor sex, male (versus female)	1.42 [1.07–1.87]	0.014	1.31 [0.98–1.76]	0.070	1.50 [1.19–1.87]	<0.001	1.34 [1.06–1.70]	0.016
Donor BMI (per 5 kg/m²)	1.12 [0.97–1.29]	0.116			1.13 [1.01–1.26]	0.040		
Cold ischemia time (per 10 hours)	1.04 [0.85–1.26]	0.715	0.99 [0.80–1.24]	0.952	1.01 [0.86–1.19]	0.887	0.98 [0.82–1.17]	0.803
Cause of death								
Stroke	Ref				Ref			
Trauma	0.64 [0.47–0.86]	0.003			0.65 [0.51–0.83]	0.001		
Anoxia	0.55 [0.33–0.91]	0.021			0.64 [0.43–0.95]	0.028		
Other	0.59 [0.27–1.26]	0.170			0.74 [0.42–1.31]	0.304		
Recipient age > 60 years	1.40 [0.99–1.97]	0.055	1.07 [0.71–1.61]	0.751	1.21 [1.10–1.33]	<0.001	1.02 [0.90–1.15]	0.726
Recipient sex, male (versus female)	1.07 [0.81–1.41]	0.655	0.95 [0.71–1.27]	0.732	0.94 [0.75–1.19]	0.620	0.85 [0.67–1.08]	0.174
Recipient BMI (per 5 kg/m²)	1.01 [0.86–1.18]	0.943			1.09 [0.96–1.24]	0.195		
Cause of ESRD								
Diabetes	Ref				Ref			
Glomerulonephritis	0.81 [0.51–1.30]	0.391			0.66 [0.46–0.95]	0.024		
Tubulo-interstitial	0.76 [0.47–1.24]	0.273			0.64 [0.44–0.92]	0.016		
Vascular	0.69 [0.30––1.62]	0.396			0.85 [0.47–1.54]	0.592		
Other	0.85 [0.41–1.75]	0.662			0.66 [0.36–1.20]	0.172		
Unknown	0.63 [0.35–1.15]	0.132			0.51 [0.32–0.82]	0.005		
number of HLA mismatchs	1.00 [0.74–1.37]	0.978			1.12 [0.88–1.44]	0.359		
First transplantation	0.55 [0.40–0.75]	<0.001	0.62 [0.44–0.86]	0.004	0.57 [0.44–0.73]	<0.001	0.54 [0.41–0.71]	<0.001
Graft rejection occurrence	3.01 [2.17–4.18]	<0.001	3.17 [2.24–4.49]	<0.001	2.33 [1.75–3.11]	<0.001	2.58 [1.90–3.49]	<0.001

Results are expressed in Hazard-Ratio (Confidence Interval 95%). GS-DC = Graft survival -death censored, GS-DNC = Graft survival - death non censored, BMI = Body Mass Index, Ref = Reference, ESRD = End-Stage Renal Disease, HLA = Human Leukocyte Antigen.

The significant risk factors of GS-DC in multivariate analysis were donor age (HR per 10 years = 1.41 [1.25–1.60]) and graft rejection occurrence (HR = 3.17 [2.24–4.49]). A first transplantation was found to be protective (HR = 0.62 [0.44–0.86]). Considering GS-DNC, in addition to the above-mentioned risk and protective factors, the donor sex (male) was also found to be a risk factor (HR = 1.34 [1.06–1.70]).

As a secondary analysis, we tested if carrying an A allele was significantly associated with a higher risk of graft failure. CC versus non-CC donors and recipients were similar (Supplemental Table 1). Carrying an A allele was also not associated with a higher risk of graft failure in uni- or multivariate analysis: GS-DC HR = 0.97 [0.77–1.21]; GS-DNC HR = 0.91 [0.69–1.20] (Supplemental Figs 1 and 2; Supplemental Table 2).

### Post-transplantation outcomes: eGFR variations

**Figure 2:**
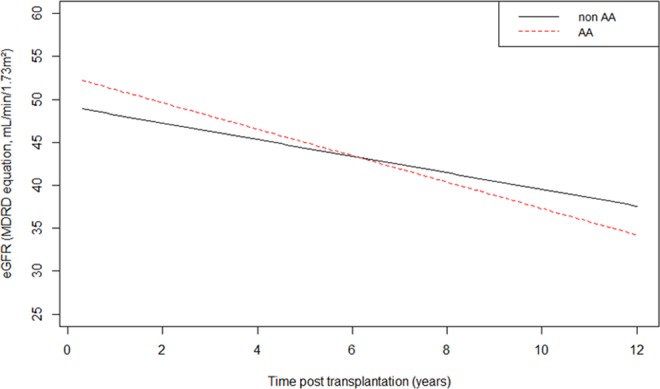
Linear mixed model for long-term estimated glomerular filtration rate comparison between *CAV1* rs4730751 single nucleotide polymorphism AA versus non-AA. n = 4785 samples. Fixed effect of AA phenotype at 3 months post transplantation eGFR: 2.95 mL/min/1.73 m² [−0.87–6.77, p = 0.13] and fixed effect of AA genotype on slope: −0.62 mL/min/1.73 m² per year [−1.33–0.13, p = 0.10].

Given the normal distribution of eGFR, this variable was not transformed. According to the Bayesian Information Criterion (BIC), the best model relation between eGFR and time was linear. Using then a linear mixed model (Fig. 2), longitudinal changes of eGFR according to the Modification of Diet in Renal Disease (MDRD) formula over time were compared between AA (n = 59) and non-AA donors (n = 764) (Supplemental Table 3). We used 4785 available values of eGFR for 823 patients with a median follow up of 4 years [1.98–7.96]. The model was adjusted on significant variables in univariate analysis, i.e. recipient sex, age, time on dialysis, Body Mass Index (BMI), previous transplantation, cold ischemia time and donor age. There was no significant difference according to *CAV1* genotype on long-term eGFRs (fixed effect of AA genotype at 3 months post transplantation eGFR: 2.95 mL/min/1.73 m² [−0.87–6.77, p = 0.13] and fixed effect of AA genotype on slope: −0.62 mL/min/1.73 m² per year [−1.33–0.13, p = 0.10]). When comparing CC donors (n = 421) versus non-*CC* donors (n = 402), there was also no difference on long-term eGFRs (fixed effect of *CC* genotype at 3 months post-transplantation eGFR: 0.21 mL/min/1.73 m² [−1.79–2.21, p = 0.83] and fixed effect of CC genotype on slope: −0.13 mL/min/1.73 m² per year [−0.52–0.26, p = 0.51]) (Supplemental Fig. 3).

### Post-transplantation outcomes: proteinuria

As a surrogate marker, we also tested if AA genotype impacted on proteinuria after transplantation. There were 7936 available values related to 695 patients with a median follow-up of 2.2 years [0.72–8.00]). Using a linear mixed model (Supplemental Table 4), longitudinal changes of urine protein/creatinine ratio were compared between AA donors (n = 47) and non-AA donors (n = 648). There was no significant difference on both baseline effect and slope effect according to *CAV1* genotype for long-term urine protein/creatinine ratio. (Supplemental Fig. 4).

### Post-transplantation outcomes: 3-months kidney biopsies

As protocolled kidney biopsies at 3 months post-transplant were systematically analyzed according to Banff classification since 2007^25^, only data for the last 394 recipients were available. Those included 25 *CAV1* AA and 369 non-AA donors. Over the tested parameters, only the proportion of globally scarred glomeruli was significantly different between AA and non-AA (4.80% versus 7.70%, p = 0.034). Of note, there was no significant difference between AA and non-AA for Banff lesions scores especially as regards markers of chronic injury, such as mesangial matrix expansion (mm score), glomerular double contours (cg score), interstitial fibrosis (ci score), or tubular atrophy (ct score). There was also no significant difference for IFTA score (Table 3).

**Table 3:** Histopathological data from systematic 3-months kidney biopsies: AA versus non AA.

	non AA (n = 369)	AA (n = 25)	p value
Sclerotic glomeruli: mean % [+/−SD]	4.80 [0.00–10.4]	7.70 [4.50–10.5]	0.034
mm score			0.131
0	301 (82.7)	16 (72.7)	
1	44 (12.1)	3 (13.6)	
2	12 (3.3)	1 (4.5)	
3	7 (1.9)	2 (9.1)	
cg score			1.00
0	355 (96.5)	25 (100)	
1	9 (2.45)	0 (0.00%)	
2	3 (0.82)	0 (0.00%)	
3	1 (0.27)	0 (0.00%)	
ci score			1.00
0	157 (42.5)	11 (44.0)	
1	162 (43.9)	11 (44.0)	
2	46 (12.5)	3 (12.0)	
3	4 (1.1)	0 (0.0)	
ct score			1.00
0	148 (40.3)	10 (41.7)	
1	172 (46.9)	11 (45.8)	
2	44 (12.0)	3 (12.5)	
3	3 (0.8)	0 (0.0)	
IFTA score			1.000
0	148 (40.1)	10 (40.0)	
1	172 (46.6)	12 (48.0)	
2	46 (12.5)	3 (12.0)	
3	3 (0.8)	0 (0.0)	
cv score			0.38
0	109 (30.4)	4 (16.0)	
1	124 (34.6)	11 (44.0)	
2	96 (26.8)	7 (28.0)	
3	29 (8.1)	3 (12.0)	
ah score			0.28
0	127 (34.5)	10 (40.0)	
1	140 (38.0)	6 (24.0)	
2	84 (22.8)	9 (36.0)	
3	17 (4.6)	0 (0.0)	

Every score was determined according to the Banff 2015 classification^30^. ah = arteriolar hyalinosis, cg = glomerular double contours, ci = interstitial fibrosis, ct = tubular atrophy, cv = vascular fibrous intima thickening, mm = mesangial matric expansion.

We also evaluated the impact of carrying an A allele (n = 208 CC and n = 186 non-CC) on kidney biopsies graded according to Banff criteria, and could not find any significant difference between the two groups (Supplemental Table 5) in particular for fibrosis.

## Discussion

The objective of our study was to evaluate the donor *CAV1* rs4730751 SNP involvement on kidney allograft outcomes. It is currently the only study which investigates precisely the association between *CAV1* rs4730751 SNP genotype and its related phenotype, through large scale clinical data, biological outcomes, and histopathological analyses. First of all, we could not find any significant impact of this polymorphism on graft survival. Secondly, one of the major strengths of our study was that we performed a longitudinal analysis of eGFR values using a mixed model, as a surrogate marker of graft survival. Again, eGFR trajectories did not differ significantly according to *CAV1* genotype. Nevertheless, we observed a trend of a higher slope decrease for AA donors, without statistical significance, which may be due to a lack of power. Finally, while using a standardized analysis with Banff classification, we could not find any significant difference on kidney fibrosis in 3-months post-transplant protocolled kidney biopsies.

Considering the previous attempts to test *CAV1* rs4730751 SNP impact on kidney allograft survival, Moore *et al*. showed for the first time a deleterious effect of this SNP^11^, when tested on two independent cohorts, the Birmingham cohort (n = 785) and the Belfast cohort (n = 679). In both cohorts, rs4730751 SNP was associated with a higher risk of graft loss (multivariate analysis AA versus non-AA: HR = 1.77 [1.08–2.90] and HR = 1.56 [1.07–2.27]). Even with a small prevalence of the AA genotype in both cohorts (respectively 7.3% and 7.1%), the risk conferred by the described association justified the authors to recommend further investigations on *CAV1* rs4730751 SNP. Indeed, the impact of caveolin-1 involvement in post transplantation outcomes could be supported with several mouse models. Knock-out mice for *CAV1* exhibited higher degree of kidney interstitial fibrosis than control mice, after unilateral ureteral obstruction challenge^26,27^. In a pro-fibrotic environment, with sustained TGFβ stimulation, caveolin-1 deficiency may be associated with an accelerated fibrotic process and impaired outcomes, as caveloae are supposed to internalize TGFβ receptor^13^. Moreover, caveolin-1 deficiency may also be associated with a different inflammatory response and a pro-fibrotic polarization of M2 macrophages after unilateral ureteral obstruction challenge in knock-out *CAV1* mice compared to wild type^27^. Unfortunately, concerning *CAV1* rs4730751 SNP, no *in vitro* or *in vivo* data are available regarding caveolin-1 functionality in patients harboring this particular intronic SNP. The study from Moore *et al*. presents several differences compared to ours which may explain the difference of results. First, fibrogenesis is a multifactorial process^16^, in which *CAV1* is known to play a role. Indeed, *CAV1* rs4730751 SNP is supposed to be involved in an accelerated fibrosis process due to impaired abilities of protection against pro-fibrotic injuries. After kidney transplantation, several events cause pro-fibrotic kidney damages, such as cold and warm ischemia times, rejection, infections like Polyomavirus-associated nephropathy, and immunosuppressive agents, especially calcineurin inhibitors^28^. Thus, there may be differences between our cohorts regarding pro-fibrotic injuries, which would explain the difference of results. Second, Moore *et al*. validated the *CAV1* rs4730751 SNP impact on two independent cohorts, including both living and brain-deceased donors, compared to our single cohort, including only brain-deceased donors. Finally, this study provided data which may be more accurate to assess long-term results, as the independent cohorts had a median follow-up of respectively 81 (54–113) and 69 (24–124) months, compared to 47.7 months (23.7–119.1) in our single cohort. Moore *et al*. provided also data on long term indication biopsies for allograft dysfunction^11^ and showed that the main cause of graft failure in the AA donor cohort was interstitial fibrosis (13 graft losses among 22 AA donors), mostly related to chronic cellular rejection (n = 6/13). On the contrary, we presented data on 3-month protocolled post-transplantation kidney biopsies, which may be too early to assess differences considering kidney fibrosis. Also, even if we could not find any effect of this SNP in our cohort, we cannot exclude that its effect could not be observed because of the differences mentioned above. After Moore and coworkers study, other teams studied the impact of *CAV1* rs4730751 SNP on kidney transplantation outcomes. Ma *et al*. presented data on *CAV1* rs4730751 SNP from 1233 kidney transplantations from Afro-American (n = 675) and European American donors (n = 558)^22^. The prevalence for AA donors was 4.00% in Afro-American donors and 7.84% in European American donors. As in our study, AA genotype was not associated with a higher risk of graft failure, but the authors were able to show interactions between other *CAV1* SNPs and *APOL1* SNPs, in particular the *CAV1* rs6466583 SNP. We cannot exclude in our study that interactions with other SNPs affecting *CAV1* sequence are underestimated. Furthermore, in our cohort ethnicity could not be collected for both donors and recipients, due to French ethical issue^29^. This could be an underlying confounder, especially considering the impact of donor *APOL1* genotype on allograft outcomes^1^. To compare Ma *et al*. study with Moore and coworkers work, the differences on survival could be due to the same reasons than ours, i.e. difference of pro-fibrotic injuries. Moreover, as in our study, the study from Ma *et al*. also had a lower median follow-up of 34.3 months (13.8–57.9) compared to Moore *et al*.^11^. It could have been interesting to provide surrogate markers, in particular long term eGFR, which would support the lack of impact of *CAV1* rs4730751 SNP in their cohort. In the same way, Van der Hauwaert *et al*. could not find any impact on graft survival in a smaller cohort (475 kidney donors, AA genotype prevalence: 7.6%)^24^, however AA genotype exhibited a significant decrease in eGFR. As in the present study, information concerning ethnicity was missing which may interfere with the results. As Van der Hauwaert *et al*.^24^, our results may suggest an impact on eGFR decrease as there was a trend of a higher slope decrease, using a linear mixed-model, for AA donors compared to non AA. Considering the low frequency of the allele, the absence of significance may be due to a lack of power. Furthermore, even if we could not find any difference on interstitial fibrosis-tubular atrophy in 3-months kidney biopsies score, we observed a significant higher percentage of globally scarred glomeruli in AA donors compared to non AA donors. This may be in line with the results of the previous studies on CAV1 genotype^11,24^, where AA donors related recipients, who had experienced a delayed graft functioning, had lower eGFRs at 3-months than non-AA donors related recipients, suggesting a decreased ability of renal recovery. In this study, AA genotype was also associated with several markers of fibrosis, with a higher risk of chronic allograft dysfunction^24^, interstitial fibrosis^24^ or vascular fibrosis^11^. AA genotype was also associated with a higher proportion of recipient showing a significant proteinuria at 5 years post-transplantation^24^. Unfortunately we could not reproduce these results with a more powerful linear mixed model.

We only focused on donor *CAV1* rs4730751 SNP effect, since evidence exists that the donor genetic background may impact on kidney transplantation outcomes^30^. Yet, considering that one of the major risk factor for graft loss in our population was graft rejection, which may be related also to the recipient genetic background, the interrelationship between donor and recipient genetic background could be relevant to investigate. Sluczanowska-Glabowska *et al*. provided data on the impact of allograft recipient *CAV1* rs4730751 SNP in a cohort of 270 kidney recipients. The prevalence of AA genotype was 7.4%^23^. There was no difference between AA *versus* non AA recipients, regarding long-term serum creatinine levels, or fibrosis index on kidney biopsies. They could also not find any difference between AA and non-AA recipients considering survivals. However, given the AA sample size, these results are probably subject to a lack of power.

Considering all these elements, assessing the real impact of *CAV1* rs4730751 SNP on kidney transplantation outcomes still remains a matter of debate. The first seminal study had a robust design and managed to observe an effect of this SNP on two independent large-sized cohorts^11^, whereas four other studies^22–24^, including ours, could not reproduce these results on strong outcomes such as graft survival. AA genotype may be associated with graft survival and an accelerated decrease of eGFR, but converging proof still remains to be produced. There may also be other specific fields of research in kidney transplantation, such as caveolin-1 genotype involvement in BK virus nephropathy, as the kidney tubular cells way of infection is thought to be caveolae-endocytosis mediated^31^. Targeting specific recipients, carrying pro-fibrotic risk factors, could also be one relevant field of research in the future.

## Patients and Methods

### Ethical statement

This observational retrospective study was performed according to the Declaration of Helsinki and the Declaration of Istanbul. No organs were procured from prisoners. As the French Biomedical Agency regulates the allocation system in France, every organ was allocated by the Agency and transplanted in Lille, France (Centre Hospitalier Régional, Lille). The protocol was certified to be in accordance with French laws by the local Institutional Review Board (Centre Hospitalier Régional, Lille), since French health authorities waived for consent requirement from deceased donors. DNA collection was registered to the French Ministry of Research under the number DC-2008-642.

### Patients

918 recipients of kidney allograft from brain-deceased donors were consecutively included from the 1st of January 2000 to the 31st of December 2016. Recipients under 18 years-old or receiving combined grafts were excluded from the study.

### Data collection

All data were collected from the CRISTAL database (French National Biomedical Agency registry) and from the recipient personal files. General demographic parameters and well-characterized risk factors of allograft failure were extracted from the database: donor age, sex, weight, height, BMI, cause of death, time of cold ischemia; and recipient age, sex, weight, height, BMI, cause of end-stage renal disease (ESRD), rank of transplantation, type of ESRD treatment (hemodialysis, peritoneal dialysis, or no treatment), number of HLA mismatches (on HLA A, B, DR), and longitudinal assessment of serum creatinine as well as eGFRs (MDRD).

### Immunosuppressive therapy

The immunosuppressive regimen followed our standard care procedure. All of the patients received an induction therapy consisting in basiliximab or thymoglobulin. Maintenance immunosuppression associated for every recipient tacrolimus, mycophenolate mofetil and steroids. Tacrolimus first doses were 0.15 mg/kg/d, and secondly adapted to tacrolimus trough level (Tac-T0). From D0 to D15, Tac-T0 targets were between 10 and 15 ng/mL. After D15, targets were between 6 and 8 ng/mL. Daily doses of mycophenolate mofetil were 750 mg twice a day. Early steroid withdrawal (day 7) was performed for non-sensitized recipients of a first renal graft.

### DNA samples and genotyping

Deceased donor DNA was extracted from lymphocytes used for the pre-transplantation cross match test. Genotyping of the *CAV1* rs4730751 SNP was performed with TaqMan allelic discrimination assays (C-29772987_10 assay) on an ABIPrism 7900HT (Life Technologies), in a 96-well plate, according to the manufacturer’s instructions. For quality control, all runs included duplicates of a null sample and of samples with known genotypes. After PCR, end-point fluorescence was measured and genotype calling was carried out using the allelic discrimination analysis module.

### Histopathologic diagnosis

From 2007, 3-month protocol biopsies after transplantation, systematically analyzed according to Banff classification^30^, were performed in our center for each kidney recipients. Tissue was embedded in paraffin, cut in 3–4 μm thick sections and stained with hematoxylin and eosin, periodic acid-Schiff, Masson’s trichrome and silver methenamine. All biopsies had adequate cortical tissue and were evaluated according to Banff criteria^30^. Fibrosis was evaluated by the IFTA score, depending on the percentage of the total kidney cortical area suffering from tubular atrophy and interstitial fibrosis (<25% = 1; 25–50% = 2; >50% = 3)^25^. Retrospectively, histopathological data were available for 394 patients.

### Statistical analysis

First, a descriptive analysis of the patients’ characteristics according to *CAV1* genotype was conducted to identify possible differences. Continuous variables are expressed as means ± standard deviations or medians (interquartile ranges), as appropriate. Categorical variables are presented as absolute numbers and percentages. Comparisons were made using the Student’s T test for quantitative variables and the Chi2 test for qualitative variables.

Second, the median overall survival and median follow-up times were estimated using Kaplan-Meier and inverse Kaplan-Meier methods, respectively. Between-group comparisons were performed using the log-rank test. Univariate followed by multivariable Cox analyses were performed to identify independent predictors of the death-censored graft survival and of the death-uncensored graft survival in two separate models.

The proportional hazard assumption was checked by log-minus-log survival curves plotting and by the scaled Schoenfeld residuals test for all covariates. When the log-linearity assumption was not met for continuous covariates, the variable was categorized in order to minimize the Bayesian information criterion (BIC).

Multivariate Cox model types were built by including all the covariates that were associated in univariate analyses, using a p < 0.20 threshold for selection, and suppressing redundant covariates. Characteristics which are known to impact long term survival were also maintained in the final multivariate models regardless of the univariate significance (i.e. recipient and donor age, sex, and BMI, cold ischemia time, donor cause of death, cause of ESRD, HLA mismatch and previous transplantation).

Third, a linear mixed model estimated by Restricted Maximum Likelihood was used to compare longitudinal changes in eGFR and urine protein/creatinine ratio according to *CAV1* genotype over time, beginning at 3 months post transplantation. The *CAV1* genotype was treated as a fixed effect associated to two random effects for baseline value and slope. If the dependent variable was not normally distributed, we considered a relevant transformation. We then chose the best fit model for eGFR variations over time on the basis of BIC values.

Univariable analyses were conducted using three effects for each variable: on baseline value, slope (interaction with time) and *CAV1* genotype. Among these parameters, those which were not significant (p > 0.20) were removed. If the association on the slope was significant, the corresponding association on baseline value was also considered. Finally, the selected significant variables were further analyzed in a multivariate linear mixed model to determine those acting independently (backward selection procedure, p < 0.05). The normal distribution of random effect on intercept, random effect on slope, residuals and homoscedasticity assumption were graphically assessed. Finally, chronic allograft injury parameters assessed on the 3 months post-transplantation systematic kidney biopsies were compared between groups of AA and non-AA genotype patients.

All analyses were performed using the 3.5.1 version of the R software with “nlme” and “survival” packages.

## Supplementary information

Supplementary Tables and Figures
