# Supplementary material for: Caveolin-1 rs4730751 single-nucleotide polymorphism may not influence kidney transplant allograft survival
Source: Sci Rep. 2019 Oct 29;9:15541. doi: 10.1038/s41598-019-52079-8 (PMC6820546; doi:10.1038/s41598-019-52079-8)
Supplement: Supplementary file 1 — Supplementary Tables and Figures [file 41598_2019_52079_MOESM1_ESM.pdf]

# Caveolin-1 rs4730751 single-nucleotide polymorphism does not influence kidney transplant allograft survival.

Mehdi Maanaoui[1], MD, Rémi Lenain[1], MD, Aghilès Hamroun[1], MD, Cynthia Van der Hauwaert[2-8], PhD, Benjamin Lopez[3], MD, Jean-Baptiste Gibier[4,5], MD, Marie Frimat[1,6], MD, PhD, Grégoire Savary[2], PhD, Benjamin Hennart[7], PharmD, Romain Larrue[7], PharmD, Nicolas Pottier[2,7], PharmD, PhD, Franck Broly[7], MD, PhD, François Provôt[1], MD, Marc Hazzan[1], MD, PhD, François Glowacki*[1,2], MD, PhD, Christelle Cauffiez[2], PhD.

1. Service de Néphrologie, CHU Lille, F-59000, Lille, France.
2. Univ. Lille, EA4483, F-59000, Lille, France
3. Service d'Immunologie, CHU Lille, F-59000, Lille, France
4. Institut de Pathologie, CHU Lille, F-59000, Lille, France
5. Univ. Lille, INSERM UMR1172, F-59000, Lille, France
6. Univ. Lille, INSERM UMR995, F-59000, Lille, France
7. Service de Toxicologie et Génopathies, CHU Lille, 59000, Lille, France
8. Département de la Recherche en Santé, CHU Lille, F-59000, Lille, France

*Corresponding author contact information* : Pr François Glowacki, Service de Néphrologie, Hôpital Huriez, CHRU de Lille, 59037 Lille, France. Tel: +33 20444034 Email: francois.glowacki@chru-lille.fr

**Supplemental Table 1: Baseline donors and recipients characteristics according to *CAV1* CC and non-CC genotype.**

| Characteristics | | **CC** (n=471) | **non CC** (n=447) | *p* value |
|---|---|---|---|---|
| Donor sex, male (versus female) | | 307 (65.2) | 288 (64.4) | 0.87 |
| Donor age: mean (SD) | | 48.0 (16.4) | 49.5 (16.1) | 0.17 |
| BMI donor: mean (SD) | | 26.1 (5.4) | 25.7 (5.0) | 0.24 |
| Cause of death | | | | 0.82 |
| | Stroke | 231 (49.0) | 214 (47.9) | |
| | Trauma | 155 (32.9) | 156 (34.9) | |
| | Anoxia | 67 (14.2) | 64 (14.3) | |
| | Other | 18 (3.8) | 13 (2.9) | |
| Cold ischemia time (minutes): mean (SD) | | 1113 (397) | 1161 (404) | 0.069 |
| Recipient sex, male (versus female) | | 288 (61.1) | 286 (64.0) | 0.41 |
| Recipient age: mean (SD) | | 50.5 (13.1) | 50.3 (13.3) | 0.81 |
| BMI recipient: mean (SD) | | 24.8 (4.6) | 24.9 (4.5) | 0.66 |
| Number of previous grafts | | | | 0.62 |
| | 0 | 382 (81.1) | 372 (83.2) | |
| | 1 | 77 (16.3) | 61 (13.6) | |
| | 2 | 11 (2.3) | 12 (2.7) | |
| | 3 | 1 (0.2) | 2 (0.4) | |
| Cause of ESRD | | | | 0.09 |
| | Diabetes | 34 (7.22) | 47 (10.5) | |
| | Glomerulonephritis | 152 (32.3) | 155 (34.7) | |
| | Tubulo-interstitial | 170 (36.1) | 132 (29.5) | |
| | Vascular | 20 (4.2) | 31 (6.9) | |
| | Others | 26 (5.5) | 24 (5.4) | |
| | Unknown | 69 (14.6) | 58 (13.0) | |
| Number of HLA mismatch (HLA A, B, DR, DQ): mean (SD) | | 3.9 (1.2) | 3.9 (1.2) | 0.64 |

Except where indicated otherwise, values were the number (%). SD= standard deviation, BMI= Body Mass Index, ESRD= End-stage renal disease, HLA= Human Leukocyte Antigen.

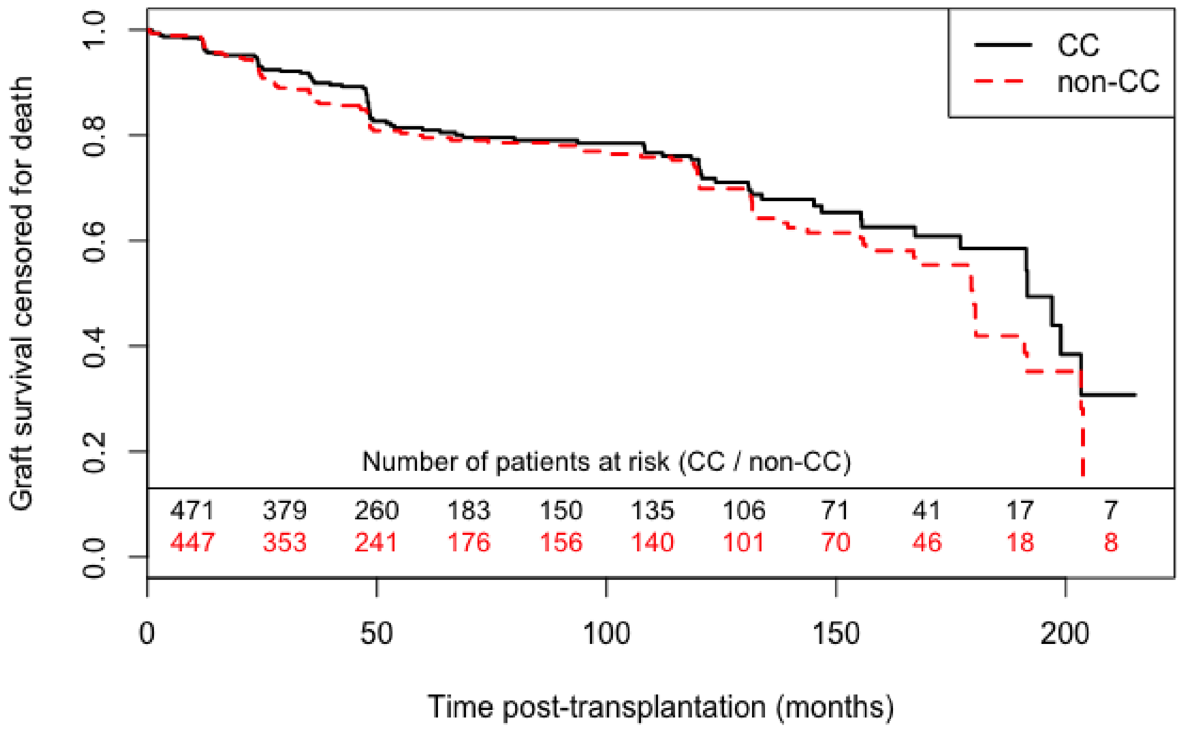


**Supplemental Figure 1: Kaplan-Meier estimates for graft-survival censored for death: CAV1 rs4730751 single nucleotide polymorphism CC versus non-CC**. Log-rank test: p=0.02

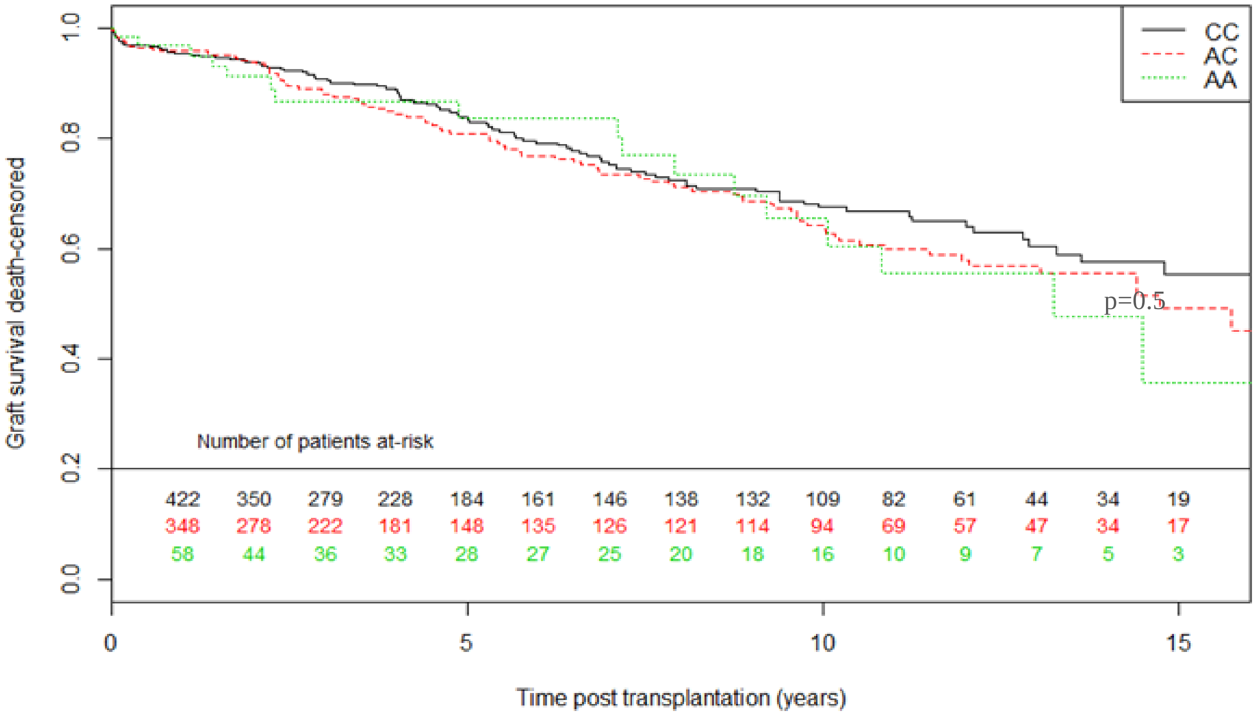


**Supplemental Figure 2: Kaplan-Meier estimates for graft-survival censored for death: CAV1 rs4730751 single nucleotide polymorphism AA versus AC versus CC**. Log-rank test: p=0.5.

**Supplemental Table 2. Multivariable Cox model for graft survival.**

| | GS-DC | | GS-DNC | |
|---|---|---|---|---|
| Variable | Multivariable | p value | Multivariable | p value |
| CAV1 genotype CC (versus non CC) | 0.97 [0.77 - 1.21] | 0.772 | 0.91 [0.69 - 1.20] | 0.493 |
| Donor age (per 10 years) | 1.30 [1.18 - 1.44] | < 0.001 | 1.41 [1.25 - 1.60] | < 0.001 |
| Donor sex, male (versus female) | 1.34 [1.06 - 1.70] | 0.014 | 1.32 [0.98 - 1.76] | 0.065 |
| Cold ischemia time (per 10 hours) | 0.98 [0.82 - 1.17] | 0.802 | 0.99 [0.80 - 1.24] | 0.956 |
| Recipient age (per 10 years) | 1.02 [0.90 - 1.15] | 0.775 | 0.80 [0.69 - 0.93] | 0.003 |
| Recipient sex, male (versus female) | 0.85 [0.67 - 1.08] | 0.181 | 0.95 [0.71 - 1.27] | 0.749 |
| First transplantation | 0.55 [0.42 - 0.72] | < 0.001 | 0.62 [0.44 - 0.86] | 0.004 |
| Graft rejection occurrence | 2.55 [1.88 - 3.45] | < 0.001 | 3.12 [2.20 - 4.42] | < 0.001 |

Results are expressed in Hazard-Ratio (Confidence Interval 95%). GS-DC= Graft survival - death censored, GS-DNC= Graft survival - death non censored, BMI= Body Mass Index, ESRD= End-Stage Renal Disease, HLA = Human Leukocyte Antigen.

**Supplemental Table 3.** Mixed model analysis for longitudinal estimated glomerular filtration rate changes between AA and non-AA donors related recipients.

| | Association with GFR (baseline effect) | | | Association with GFR evolution (slope effect) | | |
|---|---|---|---|---|---|---|
| | Coefficient | CI95% | p-value | Coefficient | CI95% | p-value |
| Intercept | 88.02 | 81.09 ; 94.95 | < 0.01 | | | |
| Time (per year) | | | | -2.26 | - 3.31 ; -1.22 | < 0.01 |
| *Cav1* AA versus non AA | 2.95 | -0.78 ; 5.78 | 0.13 | -0.61 | -1.35 ; 0.13 | 0.10 |
| Male recipient | 2.40 | 0.34 ; 4.46 | 0.02 | 0.71 | 0.30 ; 1.12 | < 0.01 |
| Recipient age (per 10 years) | 0.04 | -1.04 ; 1.12 | 0.94 | 0.32 | 0.16 ; 0.48 | < 0.01 |
| Donor age (per 10 years) | -5.71 | -6.53 ; -4.89 | < 0.01 | | | |
| Second transplantation | 0.02 | -2.74 ; 2.78 | 0.98 | -0.61 | -1.15 ; -0.06 | 0.03 |
| Recipient BMI (per 5 $kg/m^2$) | -1.75 | -2.87 ; -0.63 | < 0.01 | | | |
| Cold ischemia time (per 10 hours) | -0.03 | -0.055 ; -0.005 | 0.03 | -0.005 | -0.01 ; -0.001 | 0.04 |

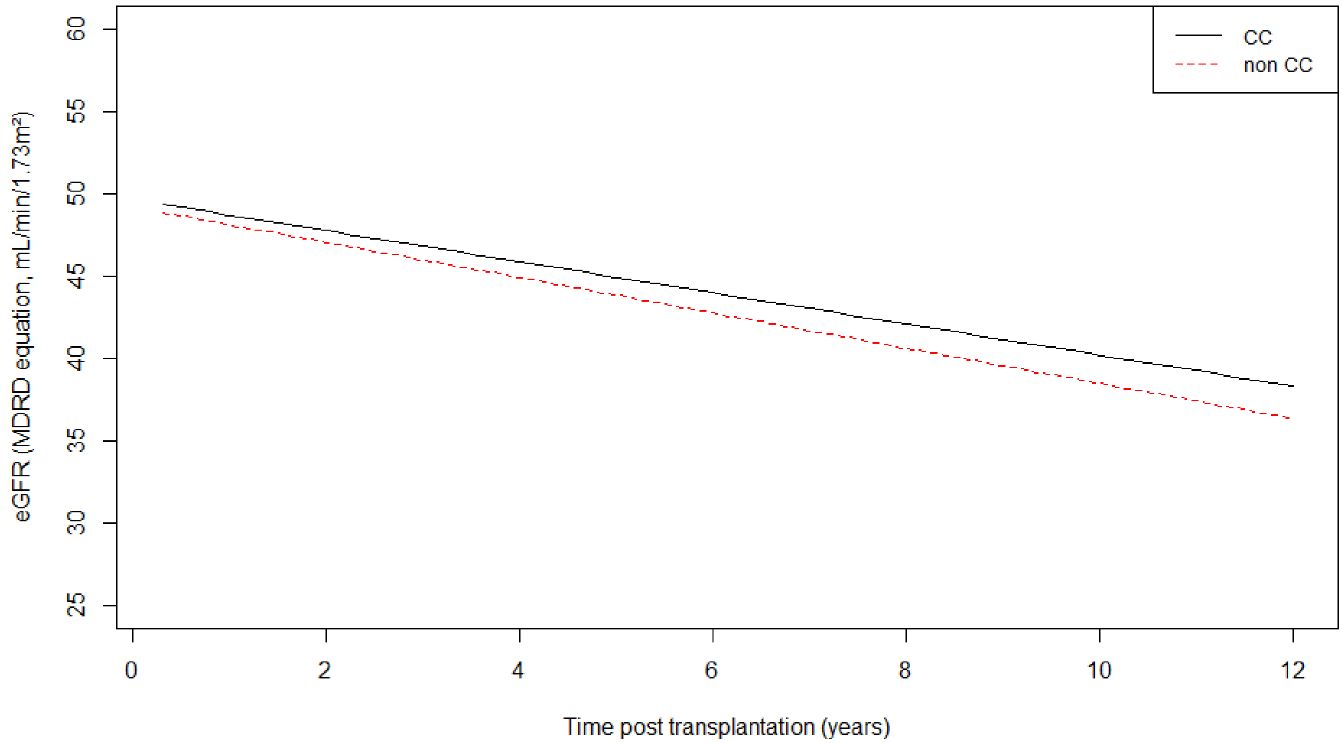


**Supplemental Figure 3: Linear mixed model for long-term estimated glomerular filtration rate comparison between CAV1 rs4730751 single nucleotide polymorphism CC versus non-CC.** n=4785 samples. Fixed effect of CC genotype at 3 months post transplantation eGFR: 0.21 mL/min/1.73m² [-1.79 – 2.21, p = 0.83] and fixed effect of CC genotype on slope: -0.13 mL/min/1.73m² per year [-0.52 – 0.26, p = 0.51]

**Supplemental Table 4.** Mixed model analysis for longitudinal logarithmic urine protein/creatinine ratio changes.

| | Association with baseline urine protein/creatinine ratio (baseline effect) | | | Association with urine protein/creatinine ratio evolution (slope effect) | | | |
|---|---|---|---|---|---|---|---|
| | Coefficient | CI95% | *p* value | Coefficient | CI95% | *p* value | Overall *p* value |
| Referential value | | | | | | | < 0.001 |
| - Intercept / Time | -1.4855 | [-1.5662; -1.4047] | < 0.001 | -0.0339 | [-0.0921; 0.0242] | 0.25 | |
| - Quadratic time | | | | -0.0014 | [-0.0146 ; 0.0118] | 0.84 | |
| - Cubic time | | | | 0.0020 | [0.0011 ; 0.0028] | < 0.001 | |
| *Cav1* AA versus non AA | | | | | | | 0.36 |
| - Intercept / Time | -0.2270 | [-0.5272; 0.0732] | 0.14 | 0.1965 | [-0.0077 ; 0.4007] | 0.06 | |
| - Quadratic time | | | | -0.0335 | [-0.0782 ; 0.0112] | 0.14 | |
| - Cubic time | | | | 0.0016 | [-0.0012 ; 0.0043] | 0.26 | |

Time is expressed as a continuous variable in years.
Time is used as a third order polynomial to account for a changing effect of time.
Also, estimated urine protein/creatinine ratio is computed as logarithmic urine protein/creatinine ratio to take in account its non-normal distribution.
Thus, , estimated urine protein/creatinine ratio can be estimated by this formula for *Cav1* AA :
$\exp(-1.4855 - 0.0339*[time] - 0.0014*[time]^2 + 0.0020*[time]^3 - 0.2270 + 0.1965*[time] - 0.0335*[time]^2 + 0.0016*[time]^3)$
and for *Cav1* non AA :
$\exp(-1.4855 - 0.0339*[time] - 0.0014*[time]^2 + 0.0020*[time]^3)$

The overall *p* value were computed by the likelihood ratio test.

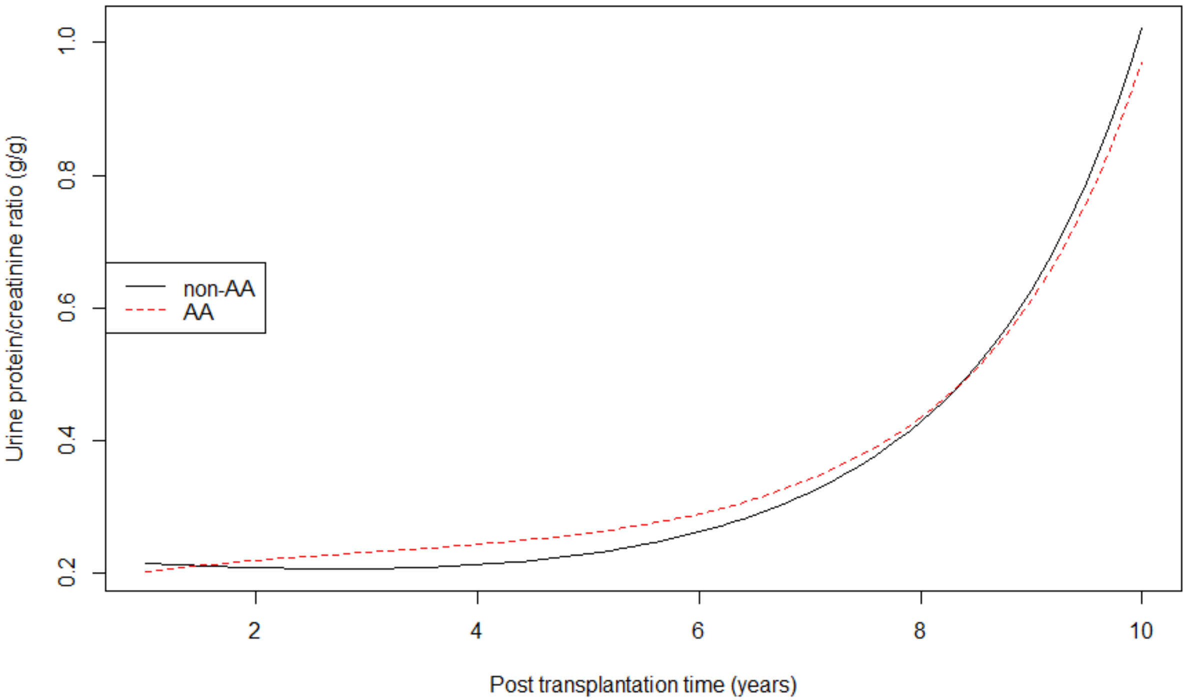


**Supplemental Figure 4: Linear mixed model for long-term urine protein/creatinine ratio comparison between CAV1 rs4730751 single nucleotide polymorphism AA versus non-AA.** n=7936 samples. Overall p-value for AA versus non-AA on baseline = 0.14. . Overall p-value for AA versus non-AA on slope = 0.36.

**Supplemental Table 5. Histopathological data from systematic 3-months kidney biopsies: CC versus non CC**.

| | | **CC** (n=208) | **non CC** (n=186) | p value |
|---|---|---|---|---|
| Sclerotic glomeruli: mean % [+/- SD] | | 20.0 [14.0 - 25.0] | 18.0 [14.0 - 23.0] | 0.71 |
| mm score | | | | 0.47 |
| | 0 | 174 (84.5) | 143 (79.4) | |
| | 1 | 22 (10.7) | 25 (13.9) | |
| | 2 | 7 (3.4) | 6 (3.3) | |
| | 3 | 3 (1.5) | 6 (3.3) | |
| cg score | | | | 0.95 |
| | 0 | 200 (96.6) | 180 (96.8) | |
| | 1 | 5 (2.4) | 4 (2.1) | |
| | 2 | 1 (0.5) | 2 (1.1) | |
| | 3 | 1 (0.5) | 0 (0.00) | |
| ci score | | | | 0.79 |
| | 0 | 89 (42.8) | 79 (42.5) | |
| | 1 | 88 (42.3) | 85 (45.7) | |
| | 2 | 29 (13.9) | 20 (10.8) | |
| | 3 | 2 (1.0) | 2 (1.1) | |
| ct score | | | | 0.68 |
| | 0 | 83 (40.3) | 75 (40.5) | |
| | 1 | 94 (45.6) | 89 (48.1) | |
| | 2 | 28 (13.6) | 19 (10.3) | |
| | 3 | 1 (0.5) | 2 (1.1) | |
| IFTA score | | | | 0.56 |
| | 0 | 83 (39.9) | 75 (40.3) | |
| | 1 | 94 (45.2) | 90 (48.4) | |
| | 2 | 30 (14.4) | 19 (10.2) | |
| | 3 | 1 (0.5) | 2 (1.1) | |
| cv score | | | | 0.87 |
| | 0 | 59 (29.1) | 54 (30.0) | |
| | 1 | 74 (36.5) | 61 (33.9) | |
| | 2 | 55 (27.1) | 48 (26.7) | |
| | 3 | 15 (7.4) | 17 (9.4) | |
| ah score | | | | 0.11 |
| | 0 | 74 (35.7) | 63 (33.9) | |
| | 1 | 67 (32.4) | 79 (42.5) | |
| | 2 | 54 (26.1) | 39 (21.0) | |
| | 3 | 12 (5.8) | 5 (2.7) | |

Every score was determined according to the Banff 2015 classification[29]. ah= arteriolar hyalinosis, cg= glomerular double contours, ci= interstitial fibrosis, ct= tubular atrophy, cv= vascular fibrous intima thickening, mm= mesangial matric expansion
